# 4063 Glycemic control in a weight management-focused group medical visits (WM/GMV) intervention: examining the moderating effects of body mass index (BMI)

**DOI:** 10.1017/cts.2020.130

**Published:** 2020-07-29

**Authors:** Elizabeth Kobe, Cynthia J. Coffman, Amy S. Jeffreys, William S. Yancy, Jennifer Zervakis, David E. Edelman, Matthew J. Crowley

**Affiliations:** 1 Duke University; 2 Durham VA Medical Center, Duke University; 3 Durham VA Medical Center

## Abstract

OBJECTIVES/GOALS: The impact of baseline BMI on glycemic response to group medical visits (GMV) and weight management (WM)-based interventions is unclear. Our objective is to determine how baseline BMI class impacts patient responses to GMV and interventions that combine WM/GMV. METHODS/STUDY POPULATION: We will perform a secondary analysis of Jump Start, a randomized, controlled trial that compared the effectiveness of a GMV-based low carbohydrate diet-focused WM program (WM/GMV) to traditional GMV-based medication management (GMV) on diabetes control. The primary and secondary outcomes will be change in hemoglobin A1c (HbA1c) and weight at 48 months, respectively. Study participants will be stratified into BMI categories defined by BMI 27-29.9kg/m^2^, 30.0-34.9kg/m^2^, 35.0-39.9kg/m^2^, and ≥40.0kg/m^2^. Hierarchical mixed models will be used to examine the differential impact of the WM/GMV intervention compared to GMV on changes in outcomes by BMI class category. RESULTS/ANTICIPATED RESULTS: Jump Start enrolled 263 overweight Veterans (BMI ≥ 27kg/m^2^) with type 2 diabetes. At baseline, mean BMI was 35.3 and mean HbA1c was 9.1. 14.5% were overweight (BMI 27–29.9) and 84.5% were obese (BMI ≥ 30). The proposed analyses are ongoing. We anticipate that patients in the higher BMI obesity classes will demonstrate greater reductions in HbA1c and weight with the WM/GMV intervention relative to traditional GMV. DISCUSSION/SIGNIFICANCE OF IMPACT: This work will advance the understanding of the relationship between BMI and glycemic response to targeted interventions, and may ultimately provide guidance for interventions for type 2 diabetes.

